# Human Cell Organelles in SARS-CoV-2 Infection: An Up-to-Date Overview

**DOI:** 10.3390/v14051092

**Published:** 2022-05-19

**Authors:** Anna Gorący, Jakub Rosik, Bartosz Szostak, Łukasz Ustianowski, Klaudia Ustianowska, Jarosław Gorący

**Affiliations:** 1 Independent Laboratory of Invasive Cardiology, Pomeranian Medical University, 70-204 Szczecin, Poland; ania.goracy@gmail.com (A.G.); jaroslaw.goracy@pum.edu.pl (J.G.); 2 Department of Clinical and Molecular Biochemistry, Pomeranian Medical University, 70-204 Szczecin, Poland; 3 Department of Physiology, Pomeranian Medical University, 70-204 Szczecin, Poland; bartszost1@gmail.com (B.S.); l.ustianowski@gmail.com (Ł.U.); ustianowska.k@gmail.com (K.U.); 4 Department of Chemistry, The University of Chicago, Chicago, IL 60637, USA

**Keywords:** COVID-19, SARS-CoV-2, ACE2, TMPRSS2, mitophagy, autophagy

## Abstract

Since the end of 2019, the whole world has been struggling with the life-threatening pandemic amongst all age groups and geographic areas caused by Severe Acute Respiratory Syndrome Coronavirus (SARS-CoV-2). The Coronavirus Disease 2019 (COVID-19) pandemic, which has led to more than 468 million cases and over 6 million deaths reported worldwide (as of 20 March 2022), is one of the greatest threats to human health in history. Meanwhile, the lack of specific and irresistible treatment modalities provoked concentrated efforts in scientists around the world. Various mechanisms of cell entry and cellular dysfunction were initially proclaimed. Especially, mitochondria and cell membrane are crucial for the course of infection. The SARS-CoV-2 invasion depends on angiotensin converting enzyme 2 (ACE2), transmembrane serine protease 2 (TMPRSS2), and cluster of differentiation 147 (CD147), expressed on host cells. Moreover, in this narrative review, we aim to discuss other cell organelles targeted by SARS-CoV-2. Lastly, we briefly summarize the studies on various drugs.

## 1. Introduction

A sequence of severe atypical viral respiratory infections occurred in the last weeks of 2019 in Wuhan, in the Chinese province of Hubei [1], spreading rapidly through droplets and direct contact [2]. Since then, the whole world has struggled with a new enveloped spherical virus [3,4,5]. SARS-CoV-2 was found to be similar to the coronavirus responsible for the outbreak of SARS [6,7]. These viruses share ~80% identity in RNA sequence [8,9]. Hence, the novel infection, Coronavirus Disease 2019 (COVID-19), has become a global health threat, and in March 2020 the World Health Organization (WHO) declared it a global pandemic [4]. To date (as of 1 May 2022), the WHO has reported over 500 million confirmed COVID-19 cases and more than 6 million deaths worldwide [10].

Every day at the turn of 2019–2020 brought us new data about this deadly virus, due to day and night research conducted in laboratories. SARS-CoV-2 belongs to the positive-sense, single-strained RNA β-coronaviruses, the Coronaviridae family, mainly found as pathogens in birds and mammals [3,4,9,11,12]. This novel coronavirus is similar to another virus recorded in recent history—Middle East Respiratory Syndrome Coronavirus (MERS-CoV), causing MERS. Moreover, SARS-CoV, SARS-CoV-2, and MERS-CoV have a similar mechanism of entry into the cell using receptors for Angiotensin II (AngII) [12,13]. Its genome is prone to random mutations [14,15,16]. SARS-CoV-2 poses 20 proteins, out of which 4 are structural and others are important for transcription and replication [17]. SARS-CoV-2’s genetic diversity affects its transmissibility, virulence, and severity of clinical manifestation, leading to outbreaks of several variants of concern (VOC) that has caused a rise in reported cases, hospitalization rates, and deaths around the world [18]. With the emergence of novel variants, the mechanism of viral entry and host cell organelles’ involvement change.

Tremendous efforts from clinicians worldwide has revealed the clinical presentation of the novel disease. Like other CoVs, SARS-CoV-2 infection severity varies from mild, self-limiting respiratory distress to severe respiratory failure or even sepsis and death [14,19]. Usually, the infection amongst the young and healthy is almost asymptomatic. However, the course of the disease tends to be fatal in the elderly and chronically ill patients [20,21]. Anosmia, cough, fever, weakness, myalgia, or difficulty breathing appear to be common symptoms [22,23,24,25,26]. Sometimes abdominal discomfort and diarrhea or neurological presentation, including delirium, might be recorded [11,23]. In critical patients, the infection might present as disseminated intravascular coagulation (DIC) or acute kidney injury (AKI) [27,28]. Infection of the lower respiratory tract might present as viral pneumonia, Severe Acute Respiratory Infection (SARI), or even progresses to multiple organ failure (MOF) [26,27]. Lung damage during COVID-19 is visible in radiological imaging most often as patchy ground-glass opacities [25,29].

Based on the above points, in the current narrative review we aim to present a summary of research concerning host cell organelles attacked by SARS-CoV-2. This selected literature review is based on in-depth analysis and selection of articles in terms of their credibility and relevance for its specific purpose.

## 2. Cell Membrane & Golgi Apparatus

### 2.1. Host Cell Entry Mechanism

The process of SARS-CoV-2 entry into the host cells is dependent on the viral Spike (S) glycoprotein. The S protein is a homotrimer, divided into two subunits, S1 and S2. The first is responsible for binding to a receptor on the surface of the host cells, while the second connects the S protein to the membrane surface, and is crucial for the fusion of membranes [30,31]. The protein is spread on the surface of the virion, creating the crown-like image in electron micrographs [32]. The S1 subunit is built of four domains: the N-terminal (NTD), receptor-binding (RBD), and two C-terminal domains (CTDs) [32,33]. The role of NTD in cell entry is yet to be established. However, NTDs in animal coronaviruses are known to promote the binding to a receptor or act as stabilization factors [34]. The RBD domain contains a receptor-binding motif responsible for the direct interaction with the receptor [35]. The connection relies on the proper conformation of RBD, which is possible due to conformation modifications of all other domains. The CTDs are key domains that stabilize the S protein structural changes needed for adequate interaction with receptor and membrane fusion [32,36].

Similar to SARS-CoV, the novel CoV uses the angiotensin converting enzyme 2 (ACE2) as the receptor to invade the host cells [37]. The ACE2 is physiologically part of the renin-angiotensin-aldosterone system (RAAS) and is involved in blood pressure and electrolyte levels regulation. The vasodilatory and anti-inflammatory effects of ACE2 are exerted by converting angiotensin II into angiotensin (1–7) [38,39]. The expression patterns of ACE2 in human tissues is similar to SARS-CoV-2 target cells. The upper respiratory tract, which is predominately the place of viral replication in an early stage of infection, is characterized by a high level of ACE2 expression. However, the highest levels of expression of ACE2 were found in the intestine, colon, kidney, or heart muscle, which explain COVID-19 organ complications. Although COVID-19 can lead to respiratory failure, the expression of ACE2 in the lung is limited to type II alveolar cells, emphasizing the other factors contributing to viral invasion [40,41,42].

**Figure 1:**
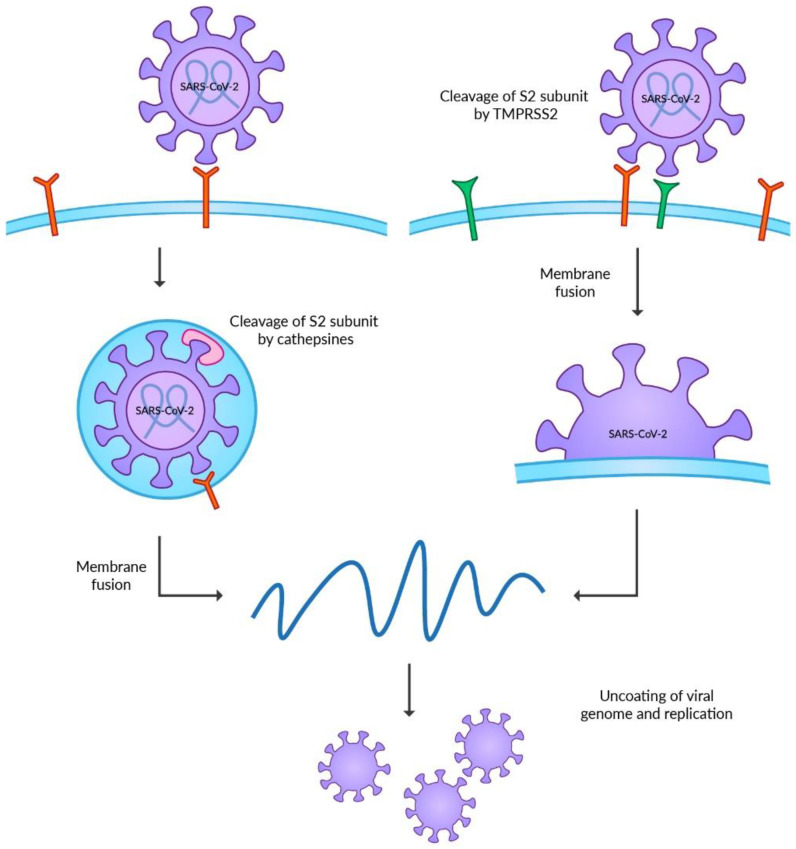
SARS-CoV-2 can enter the host cell via two separate mechanisms. The endosomal entry pathway uses cathepsins to catalyze the modification of the S2 subunit of Spike protein. TMPRSS2 is required in the surface entry mechanisms to cleavage the S2 subunit. Both courses enable membrane fusion and cell invasion.

The invasion of the host cells requires post-translational modifications of the S protein (Figure 1). Hence, the final energetic state can overcome the repulsion force between the cell membrane and the virus. The S protein undergoes two steps of cleavages before achieving proper conformation [43]. Unlike other CoVs, the first step is catalyzed by the furin or furin-like proteases during the maturation of the virus in the Golgi apparatus of the host cells. This modification in the junction between S1 and S2 subunits is required for further cleavage on the cell surface [32,44,45]. The role of this modification is still unknown, but the retention of this site, which could destabilize the S protein, emphasizes its unrevealed but essential function in the viral life cycle [46]. Furthermore, recent studies highlight the role of the furin cleavage site in the transmission of SARS-CoV-2 among ferrets [47].

Two separate pathways of host cell invasion can occur after connecting with ACE2 on the host cell membrane. SARS-CoV-2 prefers the cell surface entry; however, it requires the presence of the transmembrane serine protease 2 (TMPRSS2) [32,48]. The physiological role of the enzyme remains unknown [49]. The modifications of viral proteins by TMPRSS2 are required in the invasion process of other viruses, such as influenza viruses, MERS-CoV or SARS-CoV [50,51,52]. The TMPRSS2 is present mainly on the mucosal surface of the gastrointestinal, urogenital, and respiratory tracts [53]. The co-expression of TMPRSS2 and ACE2 was reported in the bronchial epithelium and colon, highlighting the target cells of SARS-CoV-2 [32,37]. The second pathway of cell entry is associated with the formation of endosomes. This mechanism may occur when the expression level of TMPRSS2 on the membrane surface is insufficient. In such a case, the process of endocytosis mediated by clathrin occurs, leading to the internalization of the virus connected to ACE2 [54]. The S protein could undergo the second cleavage catalyzed by cathepsins in the endosome [30,48]. This family of non-specific proteases is a vital factor in protein degradation in lysosomes and endosomes [55]. Viruses during entry into the host cells widely use cathepsins. The best known are the functions of cathepsin B in Ebola virus entry [56,57] and cathepsin L in SARS-CoV and SARS-CoV-2 entry [48,58,59]. Before the emergence of now globally dominating Omicron VOC, SARS-CoV-2 was believed to have a low dependency on the endosomal entry pathway, suggesting the limited efficacy of endosomal enzyme inhibitors on COVID-19 treatment. However, recent studies on Omicron VOC reported the higher ability of the virus to enter the host cell independently of TMPRSS2. Moreover, the endosomal entry pathway enables the Omicron VOC to target more respiratory tract cells, increasing transmissibility [60,61,62].

Both TMPRSS2 and cathepsins catalyze the modification of the S2 subunit of S protein to trigger the fusion of the membranes. After the second cleavage, the conformation of the S2 subunit is significantly changed, and the S1 subunit dissociates. These events lead to the rapprochement of membranes and the formation of a fusion pore, which enables viral entry [32].

Multiple studies have proposed alternative host cell membrane receptors for SARS-CoV-2. Neuropilin-1 (NRP1) is a member of the non-tyrosine kinase family, which is involved as a co-receptor in regulating numerous physiological activities [63]. It is believed that furin’s cleavage of the S1–S2 subunit junction could generate the binding site for NRP1. In the human cell culture, the infectivity of SARS-CoV-2 was enhanced by NRP1 due to increased entry into human cells [64,65]. The other possible co-factor of SARS-CoV-2 cell invasion is metabotropic glutamate receptor subtype 2 (mGluR2), which binds directly to the S protein [66]. A study of the mGluR2 knockout mice reported decreased infectivity of SARS-CoV-2 among mice lacking the receptor. Furthermore, the absence of mGluR2 in lung tissues restricted the endosome entry pathway mediated by clathrin [67]. The urogenital symptoms of COVID-19 could result from viral invasion through the kidney injury molecule-1 (KIMI1). Observations reported that RBD could connect with KIMI1, leading to the rapprochement of two membranes [68]. However, further analysis of this entry pathway is needed to evaluate its exact role. Other compounds involved in SARS-CoV-2 cell entry are heat shock protein A5 (HSAP5), basigin (CD147), heparan sulfate (HS), ADAM metallopeptidase domain 17 (ADAM17), and Toll-like receptor 4 (TLR4) [66,69,70,71].

The viral entry process is a promising target for efficient therapies. Research on the exact mechanism of cell invasion could deliver novel plausible targets for COVID-19 treatment.

The inhibition of S protein is one of the evaluated therapeutic targets, as it restricts cell entry [72]. The usage of previously recommended, and approved for treatment by the Food and Drug Administration (FDA), monoclonal antibodies targeting RBD of S protein is presently restricted due to Omicron VOC resistance. However, multiple novel compounds are being developed, and 100 clinical trials are ongoing or have been completed [73,74]. Nanobodies and mini proteins that interact with S protein are also being investigated [72]. The surfactant protein D (SP-D) is physiologically involved in innate immune response, clearing the respiratory tract from pathogens and necrotic cells [66,75]. In vitro studies showed that SP-D could recognize the S protein of SARS-CoV-2 and inhibit cell invasion [76]. The observation was confirmed by Madan et al., using a recombinant SP-D fragment (rfSP-D). Treatment with rfSP-D restricted viral replication on the clinical samples and was more efficient than remdesivir [77]. The levels of SP-D were also reported to be elevated in patients with a severe course of COVID-19 compared to mild, suggesting a possible role as the biomarker of disease severity [78]. The potential beneficial effects of SP-D recombinant in COVID-19 treatment are being evaluated in a phase Ib clinical trial (NCT04659122).

The proteins involved in viral entry are another possible target of novel treatment. The ACE2, as the primary receptor for SARS-CoV-2 cell entry, has become a promising target for therapies. Monteil et al., in their in vitro study, showed the efficacy of human recombinant soluble ACE2 (hrsACE2) in COVID-19 treatment [79]. However, a phase 2 clinical trial (NCT04335136) showed no beneficial effects of hrsACE2 in COVID-19 therapy [72]. There was no significant difference in all-cause death and invasive ventilation up to 28 days of treatment between patients receiving hrsACE2 and placebo [80]. The HS that co-factors the cell invasion via ACE2 could also be targeted to restrict virus infectivity. Already available drugs such as mitoxantrone or sunitinib could inhibit HS and therefore be effective in COVID-19 treatment [70]. However, in vivo studies did not prove the efficacy of these agents.

### 2.2. TMPRSS2

**Figure 2:**
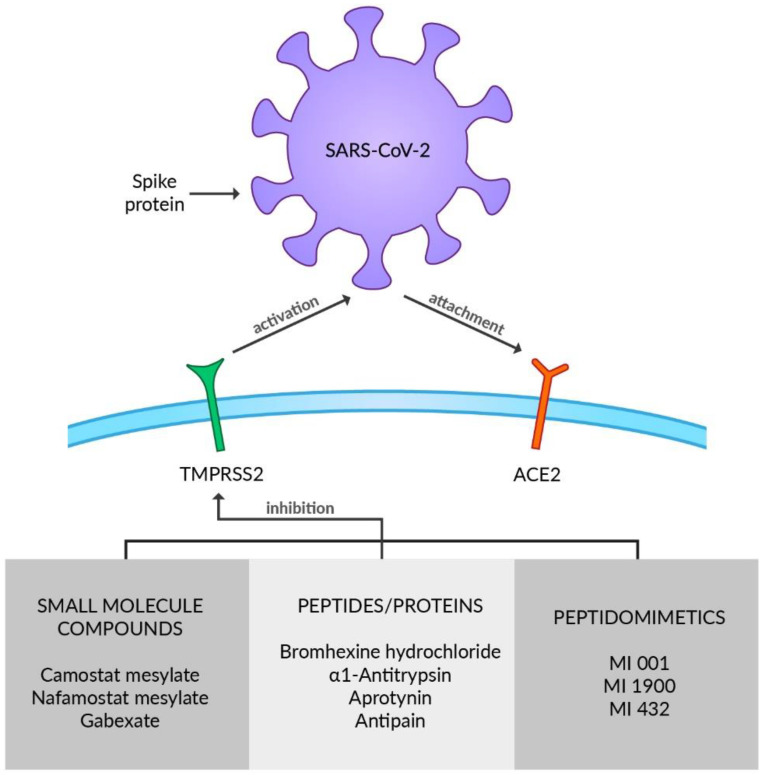
SARS-CoV-2 S protein cleavage by TMPRSS2 protease enables virus cell entry. Inhibition of TMPRSS2 activity is a promising therapeutic target in fighting SARS-CoV-2 infection.

TMPRSS2 is a member of the type 2 transmembrane serine protease (TTSP) family and is broadly expressed in the respiratory tract including nasal, tracheal, and bronchial epithelium [81,82] or type II alveolar cells [81] as well as in pancreas, colon, prostate, or salivary glands [83]. In bronchial and lung cells it is co-expressed with ACE2 [84]. Furthermore, transmembrane protease serine 4 (TMPRSS4) is reported to activate SARS-CoV-2 S proteins along with TMPRSS2, and enable viral infection of the human small intestine [85]. TTSP enzymes are constructed of an N-terminal intracellular domain, a transmembrane domain, an extracellular variable stem region, and a C-terminal serine protease domain [86]. Although the physiological aspect of TMPRSS2 remains incomprehensible, it certainly plays an essential role in viral infection by priming and activating SARS-CoV-2 S proteins [48]. The SARS-CoV-2 surface glycoprotein S must be cleaved at two specific sites by host cell proteases for the virus to be able to enter cells. Thus, potential drug targets emerge [87]. SARS-CoV-2 S protein contains two subunits: S1 with the RBD and S2 with the viral fusion machinery, which is produced as an inactive precursor [88]. The S1/S2 cleavage site of SARS-CoV-2 S is located at the interface of the subunits and has been documented to be mediated by furin [30,87]. It enables the activation of protease cleavage site S2 by the TMPRSS2 upon receptor-binding and afterward, and the membrane fusion is triggered, which is necessary for viral entry to respiratory cells [89]. Furin induces proteolytic activation on mammalian, viral, and bacterial substrates, which can lead to various diseases. Several viruses, such as Herpesvirus, Paramyxovirus, and Togavirus, use furin to activate their glycoproteins, including the S protein of COVID-19 [6]. Considered a mechanism of SARS-CoV-2 spreading, furin-wide organ distribution may be a possible explanation for the infection and corresponding symptoms [6]. Some of the already known antiviral drugs such as Indinavir, Tenofovir, Alafenamide, Tenofovir, Disoproxil, Dolutdegravir, Boceprevir, and Telaprevir may act as potential furin inhibitors that might be beneficial for COVID-19 treatment [90]. Nonetheless, further in vitro and in vivo studies need to be conducted to verify these assumptions because it has only been evaluated on the modeling level [6]. Since transcription of the *TMPRSS2* gene is regulated by androgen receptors, their antagonists may be considered as a therapeutic option against COVID-19 due to its role in downregulating TMPRSS2 [72]. However, the androgen reliance of TMPRSS2 expression in the human respiratory tract has not been fully explicated and should be verified with additional studies [91,92]. A large spectrum of TMPRSS2 inhibitors has already been reported, including small molecule compounds, peptides/proteins, and peptidomimetics (Figure 2). The drugs usually inhibit proteolytic activity; however, reducing expression or synthesis of TMPRSS2 is being considered as a therapeutic target [86]. Camostat mesylate (CM) and nafamostat mesylate (NM) are identified as serine protease inhibitors belonging to the group of small molecule compounds that demonstrate activity against TMPRSS2. CM is utilized in chronic pancreatitis and postoperative reflux esophagitis therapy in Japan [93,94]. Complementary to CM, NM is used for the treatment of pancreatitis in Japan and Korea and as an anticoagulant in disseminated intravascular coagulation (DIC) therapy [95,96]. As abnormal coagulation, DIC, and venous thromboembolism are concerned in severe cases of COVID-19, due to its various actions such as antithrombin, antiplasmin, and antitrypsin effects NM is thought to curb the pathogenicity and infectivity of SARS-CoV-2 [97]. CM not only constrains cell entry of SARS-CoV-2 by inhibiting TMPRSS2 but also decreases infectivity of other respiratory viruses such as influenza [12,98]. NM inhibited recombinant TMPRSS2 more efficiently than CM [99]. Oral administration of CM proved to be efficient improving inflammatory markers and clinical severity in COVID-19 patients with MOF during a retrospective case study [100]. Moreover, the group of COVID-19 patients treated with CM had accelerated resolution of symptoms compared to the placebo group [101]. In contrast, in a placebo-controlled clinical trial on hospitalized COVID-19 patients, Guns et al. demonstrated that CM had no substantial effect on clinical outcomes in comparison to the placebo group [102]. However, the possibility that CM treatment in a higher dose or during the very early phase of COVID-19 might be effective in lowering the risk of disease progression should be further investigated, as well as a trend toward a lower rate of ICU admission during CM therapy [102]. Clinical and radiologic improvement was experienced in preliminary uncontrolled cases of COVID-19 patients with pneumonia treated by intravenous administration of NM [103]. Treatment with NM during a phase II clinical trial in hospitalized patients with COVID-19 pneumonia requiring oxygen supplementation found no significant difference in time to clinical change between the NM and standard-of-care groups [104]. Although a quicker clinical improvement in a subgroup of high-risk COVID-19 patients requiring oxygen treatment was reported, it requires further verification [104], along with studies as to whether CM or NM caused no or only mild adverse events [102,104]. No evidence of anti-inflammatory, anticoagulant, or antiviral activity was found after applying intravenous NM therapy in hospitalized COVID-19 patients and there were adverse events in the experimental study of Quinn et al. [105]. The combination of NM and favipiravir is being examined in a clinical trial in Japan (jRCTs031200026). CM and NM are currently being tested against COVID-19 in several phase 2 and 3 clinical trials in many countries (NCT04623021, NCT04390594, NCT04483960, NCT04521296, NCT04721535, NCT04530617) [106]. Even though another small molecule protease inhibitor Gabexate is reported to inhibit TMPRSS2, the results of its antiviral activity remain inconsistent [107]. SARS-CoV-2 S-mediated entry in lung cells was not affected by Gabexate [108]. As a result, further experiments need to be conducted to establish Gabexate’s influence on protease activity and viral replication. Bromhexine hydrochloride (BHH), which is used to reduce cough in children and adults, is another drug targeting TMPRSS2 activity, making it a potential therapeutic option in COVID-19 [109,110]. A few studies have already scrutinized BHH’s effectiveness regarding SARS-CoV-2 infection or COVID-19 progression. A significant decrease in the rate of ICU admission and need for ventilation after BHH administration was established in patients with COVID-19 symptoms [110]. Prophylactic BHH intake was investigated in a non-peer-reviewed study that showed a connection between BHH prophylaxis and a reduced rate of symptomatic COVID-19 infection but showed no relevance in the lowering combined primary endpoint rate, a positive swab PCR test, or COVID-19 [111]. A non-significant trend toward better clinical recovery upon administration of BHH was reported by Li et al. [112]. BHH failed to accelerate the clinical improvement of COVID-19 patients in the Tolouian et al. study [113]. Contradictory outcomes of the experiments may come as a result of various study designs and further clinical trials are required to formulate the applicability of BHH in COVID-19. α_1_ antitrypsin (α_1_AT) is a serine protease inhibitor used in emphysema patients with α_1_AT deficiency [114]. Moreover, it is reported to moderate acute lung injury by inhibiting inflammation, neutrophil elastase activation, cell death, and coagulation [115]. α_1_AT restricts protease-mediated cellular entry of SARS-CoV-2 because of its inhibiting impact on TMPRSS2 activity [116]. The increased interleukin 6 (IL-6):α_1_AT ratio reflecting the balance between pro- and anti-inflammatory mechanisms has been related to poor outcomes in COVID-19 ICU patients [117]. The anti-SARS-CoV-2 infection, immunomodulatory and anti-inflammatory features of α_1_AT make it a promising candidate for the treatment of COVID-19 [115]. Accordingly, three clinical trials of α_1_AT for COVID-19 treatment have already been initiated (NCT04385836, NCT04495101, NCT04547140) [115]. Furthermore, aprotinin is a polypeptide obtained from the bovine lung, which is also shown to have an inhibitory effect on TMPRSS2, although direct evidence is still required [118]. The SARS-CoV-2 cytopathic effect and replication were alleviated by aprotinin in immortalized cells [106]. Combined therapy with aprotinin and favipiravir led to encouraging results in preventing progress of COVID-19 in patients requiring oxygen therapy [119]. Moreover, their hospital stays were diminished [119]. HGF activator inhibitors HAI-1 and HAI-2, transmembrane Kunitz-type protease inhibitors expressed in the prostate and the respiratory tract, and reduce TMPRSS2 activity in vitro by Ko et al. [120,121]. Along with the above proteins, the oligopeptide antipain and the protein soybean trypsin inhibitor (SBTI) inhibited TMPRSS2, the entry and genome replication of SARS-CoV-2 [122]. Meyer et al. developed a series of synthetic peptidomimetics, including compound MI-001, compound MI-1900, and compound MI-432, acting as TMPRSS2 substrate analogs and inhibitors suitable for inhibiting influenza virus activation [123]. Bestle et al. reported strong inhibition of SARS-CoV-2 replication by treatment of Calu-3 cells with MI-432 and MI-1900 [87]. Consequently, the physiological tolerability of these compounds should be considered during the future development of antiviral therapeutics [86,124].

### 2.3. CD147

**Figure 3:**
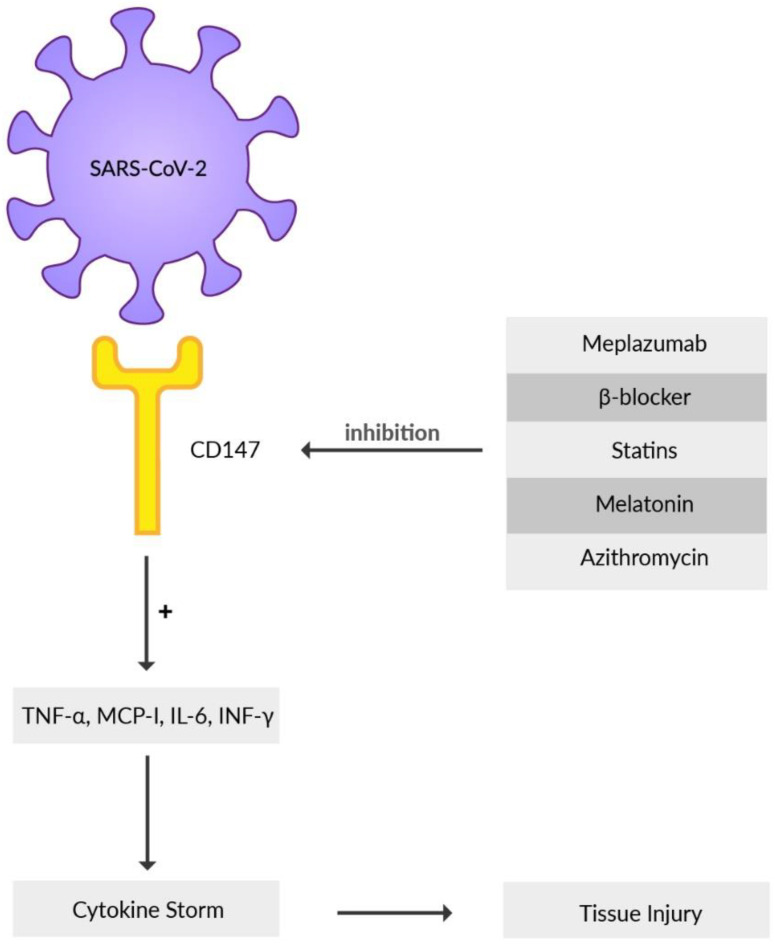
**CD147-mediated signaling pathway.** The serum cytokine increase (TNF-α, MCP-1, IL-6, and INF-γ) contributes to the cytokine storm in the host organism. CD147 inhibitors prevent SARS-CoV-2 entry into the host cell.

Basigin (BSG), an extracellular matrix metalloproteinase inducer (EMMPRIN) or cluster of differentiation 147 (CD147), is a highly glycosylated transmembrane protein that contributes to the development of tumors, invasion of the human malaria parasite Plasmodium, inflammatory processes, and infection mediated by various bacteria or viruses [125,126,127,128]. Recently, it has been reported that CD147, besides ACE2, is another receptor that the SARS-CoV-2 virus directly interacts with and subsequently invades the host cell when its S protein binds to CD147 [71]. The substantial involvement of CD147 as an alternative virus receptor for ACE2-defiant cells for SARS-CoV-2 infection was reported by Wang et al. using mouse models [71]. Immune cells such as T cells, B cells, macrophages, or natural killer cells may spread the virus from infected epithelial cells via CD147, causing local and systemic virus expansion throughout the body [129]. During SARS-CoV-2 infection, the uncontrolled release of cytokines worsens the disease course and creates a hyper-inflammatory state in the host organism [130]. CD147, by producing pro-inflammatory cytokines, such as interferon-γ (INF-γ), IL-6, monocyte-chemoattractant protein (MCP-1), and tumor necrosis factor-α (TNF-α), contributes to the inflammation (Figure 3) [131]. SARS-CoV-2 may cause oxidative stress-induced damage in erythrocytes using CD147 receptors which can provoke myocardial injury and refractory hypoxemia in COVID-19 patients requiring mechanical ventilation [132]. It is described that the increase of glucose level in blood elevates the expression of CD147 receptor on the immune cells [133]. This may be associated with COVID-19 patients with type-2 diabetes and poor blood glucose management, presenting a higher mortality rate than individuals where the blood glucose level was well preserved [134]. CD147 is expressed in high amounts in bronchial epithelial cells in patients suffering from chronic obstructive pulmonary disease (COPD) as well as in the blood of obese patients, common comorbidities concomitant with COVID-19 [135]. All these data indicate the possible involvement of CD147 in COVID-19 pathogenesis, mainly in patients with other diseases. A detailed comprehension of the interaction between CD147 and SARS-CoV-2 will provide better insight and therefore facilitate the formation of a possible COVID-19 therapy [136]. Meplazumab, a humanized anti-CD147 antibody, inhibits binding of SARS-CoV-2 S protein with CD147, which can prevent viruses from entering the host cells, creating a promising target for forming an antiviral drug [6]. Meplazumab proved to have a significant impact on the improvement of the condition and recovery rate of patients infected with COVID-19 pneumonia. This is under phase 2 clinical trials in China [137]. Other results indicate that meplazumab may accelerate the recovery of COVID-19 patients, with a good safety profile [138]. Beta-adrenergic blockers may have a beneficial influence on COVID-19 infection because of their hindering effect on CD147 expression and, as a result, inhibit the cellular entry of SARS-CoV-2 [139]. Statins should be considered a co-adjuvant therapy for COVID-19 management because of their ability to downregulate CD147 levels [140]. Azithromycin may be taken into consideration as a possible candidate for soothing the course of SARS-CoV-2 infection due to its role in interfering with CD147 receptors and preventing the invasion of Plasmodium falciparum [137]. The CD147-mediated signaling pathway is reported to be inhibited by melatonin. Furthermore, there are possible beneficial effects of melatonin as adjuvant use in COVID-19, such as anti-inflammation, anti-oxidation, and immune response regulation [141]. It may be possible to prevent the development of severe disease symptoms in patients with COVID-19 and reduce the immuno-pathology of SARS-CoV-2 infection after the active phase of the infection is over by utilizing melatonin [142]. A few trials have already demonstrated the benefits of melatonin in COVID-19 [143]. Additional experiments and clinical studies need to be conducted to confirm this speculation. Notably, some observational studies showed no association between lower mortality and a daily dose of melatonin administration in COVID-19 patients [141,144].

*N. sativa* is a herb renowned for its seeds, which present many active compounds beneficial for the immune system. Its oil was researched by Sultan et al. in 2015, who showed that *N.* *sativa* seed powder decreased oxidative stress in human cells [145]. In animal studies, it proved to be a successful medium, stimulating an immune response, lowering the cytokine storm intensity, and increasing anti-inflammatory response [146,147,148]. *N. sativa* seed can promote injured cells’ autophagy, a mechanism disrupted by the SARS-CoV-2 virus [149,150]. It is suggested that *N. sativa* oil may be used as an adjuvant therapy as its properties seem to address problems caused by the SARS-CoV-2 virus [151]. Its three main components are thymoquinone, α-hederin, and nigellidine. Thymoquinone presents antioxidative and antibacterial properties. It downregulates interferon activation and inhibits the production of pro-inflammatory cytokines [152,153,154,155]. The α-hederin molecule exhibits antihistaminic, anti-eosinophilic, antileukotriene, anti-immunoglobulin, and reduced-proinflammatory cytokines (IL-2, IL-4, IL-5, IL-6, IL-12, and IL-13) in in vitro/in vivo models [156]. It is also suggested that α-hederin and nigellidine can inhibit COVID-19 disease, but further research is needed [157].

### 2.4. DPP4

Dipeptidyl peptidase 4 (DPP4) is a serine exopeptidase whose catalytic activity is based on cleaving two residues from the N-terminal end of many peptides [158]. Its expression has been detected in many cells, such as endothelial, epithelial, and immune cells, in the lungs, spleen, pancreas, or intestine [159]. The enzyme is commonly known for its role in inactivating incretin hormones and utilization of its inhibitors in type 2 diabetes therapy [160]. Moreover, DPP4 can also act on other substrates such as cytokines, chemokines, growth factors, or processes such as apoptosis, cell adhesion, chemotaxis, and co-stimulatory function in T-cell activation [161,162]. Additionally, DPP4 inhibitors are believed to induce some anti-inflammatory effects [163]. Acknowledging COVID-19 as an inflammatory disease with diabetes as a risk factor for a severe course of the infection, it is of interest to study the relationship between clinical outcomes and use of DPP4 inhibitors [164]. Some hypotheses assumed interaction between SARS-CoV-2 S protein and DPP4, which could indicate DPP4’s part in virus cell entry together with ACE2 and lead to the application of its inhibitors for antiviral effects [165]. It was also implied that gliptins may play a role in SARS-CoV-2 main protease inhibition [166]. Both assumptions proved to be wrong in functional studies, as S protein and DPP4 do not bind, and DPP4 inhibitors do not inhibit the main protease in conducted experiments [167,168]. Some observational studies showed positive effects such as decreased mortality after using sitagliptin in patients with type 2 diabetes and likewise reduced mortality risk in COVID-19 when applying gliptin therapy before hospitalization [169,170].

On the contrary, other studies presented no significant association between DPP4 inhibitors application and the evolution of SARS-CoV-2 infection [171,172]. No decreased risk of hospital admission as well as no improvement in clinical outcomes were reported in patients with diabetes and SARS-CoV-2 treated with incretin-based therapies [173,174]. Thus far, there is no confirmed evidence of gliptin’s beneficial effect on the COVID-19 clinical course. Randomized and prospective clinical testing is necessary to assess this accurately [175].

## 3. Mitochondria

Mitochondria are regarded primarily as power generators for a cell’s metabolism. However, they take part in a number of other processes, whether physiological or pathological. They mediate in mineral homeostasis, synthesis of steroids, or even apoptosis [176,177]. Although these are crucial for the cell’s maintenance or even the homeostasis of a whole organism, their most fundamental role is to produce energy for the cell in the form of ATP. Unfortunately, that process is also targeted by many single-stranded RNA viral infections, which are known for altering the physiological pathways in favor of viral reproduction [178,179]. Hijacking ATP production enables viruses to take over a viable source of energy to sustain their own needs for replication. Mitochondria are also essential organelles in cellular defense mechanisms and viruses became able to intercept those functions and alter them to evade innate immune response [180,181]. On its outer membrane (OMM) are located various proteins such as mitochondrial antiviral signaling protein (MAVS), used in antiviral response, or voltage-dependent anion channel (VDAC), crucial for maintaining calcium levels between mitochondrial matrix (MM) and endoplasmic reticulum (ER) [182].

**Figure 4:**
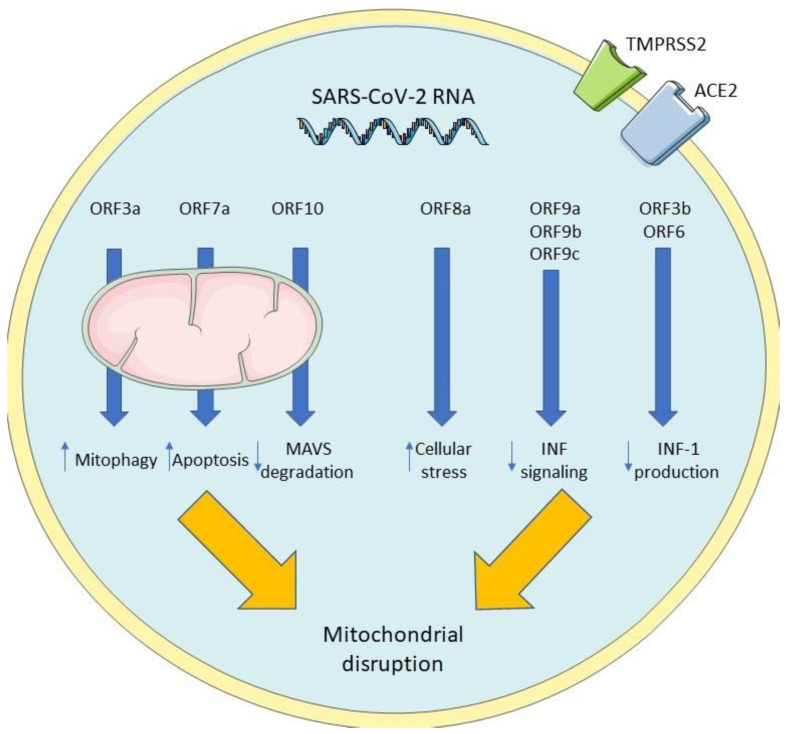
**Metabolic changes caused by SARS-CoV-2 virus in the host cell.** Open reading frames (ORFs) are responsible for various changes to the host cell metabolism, which are focused on damaging mitochondria.

In SARS-CoV-2 virus, various open reading frames (ORF), a genome sequence between the starting and ending codon which produces a functioning protein, have been found to interact with the host cell, specifically mitochondria, in order to alter its metabolism for viral infection. These proteins, grouped by the ORF that they come from, induce various changes in a host cell resulting in mitochondrial disruption [183]. Gordon et al. have successfully cloned and expressed most of the SARS-CoV-2 proteins. They also have mapped interactions between them and human proteins [184]. One of the most important is M protein, interacting with host mitochondrial proteins, which can induce cellular apoptosis [184]. ORF3a encodes a protein that targets a mitochondrial ubiquitinase responsible for mitophagy control and homeostasis. ORF3b and ORF6 inhibit INF-1 induction, helping evade cellular antiviral response [185,186]. ORF7a uses its transmembrane domain to take part in inducing apoptosis [187]. ORF8a is believed to increase reactive oxygen species (ROS) creation, oxygen consumption, cellular stress build-up and, when studied in vitro, enhanced viral replication [188]. ORF9a, 9b, and 9c are suggested to take part in inhibiting INF signaling and favoring viral replication (Figure 4) [189,190,191,192]. ORF10 increases MAVS degradation, promoting viral reproduction [193].

All proteins encoded in the SARS-CoV-2 genome aim to alter the host cell metabolism for reproducing viruses. After entering the host cell, the virus starts its replication progress in the cytoplasm. Physiologically this process is detected by TLRs and mitochondrial viral signaling systems (AVMs) [180,194]. However, SARS-CoV-2 evades these by producing a double membrane vesicle using mitochondria and ER [195]. It enables the virus to easily penetrate into the mitochondria unnoticed and further undergo a replication process, which was proven by showing the presence of the SARS-CoV-2 RNA genome in mitochondrial matrix (MM) and nucleus [180,196]. This provokes great amounts of effort in ER as it tries to maintain its normal functions, now with additional processes. This overload of ER triggers its disruption, excretion of Ca^2+^ ions and subsequently calcium ion deficiency in ER. This causes VDAC to engage in tunneling those ions into the MM, which increases the amount of ROS being produced in mitochondria. As a result, there is an increased release of interleukins 1β (IL-1β), 6, and 18, as well as TNF-α. Additionally, ORFs help avoid instant response from antiviral mechanisms. They intensify the inflammation state and slow down the response, causing the cytokine storm [183]. This process reprograms host cell metabolism into the lycolysis cycle, lowering the ATP produced by the Krebs cycle and inducing mitochondrial atrophy [197]. TNF-α causes calcium-dependent increase of mitochondrial ROS (mt-ROS), which further decreases host cell function. These changes lead to increased mitochondrial membrane permeability and release of mitochondrial DNA (mt-DNA). Together, interleukins, mt-DNA, and elevated levels of ROSs speed up cellular aging and death. Singh et al. demonstrated that lung cell lines infected with SARS-CoV-2 upregulated their genes involved in inflammatory response and downregulated genes connected to mitochondrial organization, respiration, and autophagy. This strengthened the previous study by Zhang et al., where it was shown that alveolar epithelial cells with dysfunction in mitochondria produce some of the pro-inflammatory cytokines, later shown to be elevated in COVID-positive patients [183,198]. These changes lead to tissue damages, hypoxia, and the creation of damage-associated molecular patterns (DAMPs). If not treated properly, they tend to build up and finally cause MOF in the most severe cases of untreated SARS-CoV-2 infection. Recent discoveries in biology found that mitochondria are evolutionarily conserved bacteria with many similarities shared between the two. Further research showed that some of the DAMPs are formyl peptides, which in nature are found only in bacteria. This suggested that DAMPs from mitochondria produce the same kind of response as a sepsis caused by bacterial infection, which was later proved by studies [199,200,201,202]

Mitochondrial disruption, apart from creating the changes shown above, creates other pathophysiological consequences. Mitochondrial iron stored in ferritin complexes is excreted by mitoferrin protein, causing increased levels of iron in the bloodstream. Hyper-ferritinemia is responsible for hyperinflammation and additional mt-DNA damage, exacerbating the ongoing release of inflammatory mediators, such as ILs, TNFs, and ROSs. It also shifts mitochondria to the anaerobic respiration cycle, creating vast amounts of lactate dehydrogenase (LDH) [203]. Later, these changes create a build-up of NAD^+^ molecules, which further inhibit mitochondrial metabolism. It is clear that at this point a positive-feedback loop is created, intensifying inflammatory response and speeding up aging processes and premature death of mitochondria [204].

SARS-CoV-2 also attacks mitochondria in platelets, causing their abnormal functioning [205]. This leads to their malfunction, and with no cell nucleus in platelets, they cannot be replaced. This creates dramatic changes, which in a short period of time end with platelet malfunction and increased risk of thrombus forming in blood vessels [194], consistent with the findings of studies showing that thrombocytopenia is more prominent in patients with a severe state of COVID-19 infection compared to those with mild infection.

The first drug to treat COVID-19 approved by the FDA was remdesivir. This adenine nucleoside analog interferes with viral RNA polymerization, lowering viral reproduction. However, it was shown to possess rather severe adverse effects associated with mitochondrial toxicity. Remdesivir was proven to disrupt mitochondrial polymerase and inhibit mitochondrial respiration [206,207,208]. Similarly, ribavirin was proposed to treat COVID-19 as a guanine nucleoside analog. Studies with MERS-CoV showed promising results. However, in SARS-CoV-2 patients it did not improve the mortality rate [209,210]. In later studies, ribavirin showed high toxicity, leading to mitochondrial malfunction, multiorgan dysfunction, and acidemia [211]. As the SARS-CoV-2 virus hijacks mitochondrial metabolism, it is a potential target for novel therapeutic agents. Additionally, strategies focused on improving mitochondrial function are equivalently crucial, as drugs used to treat COVID-19 present mitochondrial toxicity and may lead to further damage to patients’ health.

### 3.1. Mitophagy

Mitophagy is a physiological process that enables cells to selectively sequester imperfect mitochondria and destroy them in lysosomes, a process to maintain homeostasis [212]. Any disruption of this process can lead to mitochondria metabolic dysfunction [213,214]. It is a crucial mechanism in response to SARS-CoV-2 infection because it controls mitochondrial quality and helps to eliminate viral dsRNA. One of the important mechanisms is a Pink1/Parkin pathway. A serine-threonine kinase (Pink1) accumulates in the OMM in case of pathology and depolarization of mitochondria [215,216]. It recruits Parkin protein, with the activity of a ligase ubiquitin E3, that triggers polyubiquitination of mitochondrial substrates [215,216,217]. It then is recognized by ubiquitin-adaptive protein P62 and undergoes an autophagy process [217].

A ubiquitin-marked substrate binds with the UBA domain in P62, LIR domain in the P62 protein binds to LC3 protein enabling selective binding with a marked substrate, and Pink1 is destroyed by proteolytic enzymes [215]. Shang et al. showed that, in cells infected with SARS-CoV-2, a Pink1-Parkin-P62 pathway becomes activated and is further stimulated as time passes. They also showed that the virus can disrupt the binding of the LIR domain of P62 protein with LC3 protein. This lowers the cell’s ability to ubiquitinate seized mitochondria, which blocks mitophagy, resulting in disrupted mitochondrial homeostasis and lowered viral RNA degradation [218].

MAVS is a cell protein that takes part in the innate immunological response to viral infection. Its increased expression leads to high INF-1 level. If MAVS expression is suppressed, INF-1 production is also weakened, enabling viruses to replicate more easily and infection to develop [182]. In the SARS-CoV-2 genome, there are 14 open reading frames (ORFs) with 11 auxiliary proteins, such as ORF10 [219,220]. Xingyu et al. showed that ORF10 blocks innate antiviral response. It also promotes MAVS degradation, which enhances viral replication [193].

Mitophagy processes can be ubiquitin-dependent or independent. The pink1-Parkin pathway is ubiquitin-dependent as it phosphorylates its chains and then autophagy receptors become active, such as P62 protein [221,222]. On the other hand, the ORF10 pathway induces mitophagy without Pink1 protein. Overexpression of ORF10 proteins is a reason for LC3 protein to appear in OMM. Mitophagy receptor Nip3-like protein X (NIX) is a mitophagy specific modulator located in OMM and, when it reacts with LC3B and ORF10, it induces mitophagy. Its effect is MAVS degradation enabling viral replication. Additionally, ORF10 overexpression causes the destruction of proteins of OMM.

Oxidative stress is a factor that may initiate the mitophagy process. Thus, several antioxidants are proposed to eliminate ROS. These include caffeine, curcumin, and various vitamins, as shown in a study by Shan et al. [223]. Mitophagy can be inhibited by pharmacological inhibition of lysosomal acidification conducted in vitro by Yang et al. [224]. Likewise, inhibiting the upregulation of Parkin and PINK1 expression was shown to be a successful approach by Wu et al. [225]. Most of the previous studies concentrated on mitochondria as the virus replication point, or cellular organelles used by virus metabolic pathways, such as mitochondrial permeability transition pores (MPTP) or unfolded protein response (UPR).

UPR is a cellular response to the endoplasmic reticulum (ER) stress [226,227,228]. It activates when numerous unfolded or misfolded proteins accumulate in the lumen of the ER. Amen et al. have shown that liraglutide, an antidiabetic drug, and atorvastatin, an anticholesterolemic agent, can modulate UPR stress [229,230]. Such an effect was also presented by melatonin in studies by Almanza et al. [231].

Intracellular complex NOD-, LRR- and pyrin domain-containing protein 3 (NLRP3) acts as a detector for cellular danger signals. If one occurs, it creates an NLRP3 inflammasome, which later leads to increased release of pro-inflammatory cytokines and further to either inflammatory response or even cell death [197]. This cascade can be inhibited by glyburide and thalidomide, as shown by Xu et al. [232].

MPTP is a transmembrane complex causing the increase of mitochondrial membrane permeability. This process regulates mitochondria path during cell death. The studies of Christ et al. have shown that drugs such as haloperidol, donepezil, and dextromethorphan can act as modulators for MPTP [233,234].

### 3.2. Other Organelles

Autophagy is a catabolic process where chemical substances or organelles are broken down in a controlled manner. Substrates are sequestered into an autophagosome. Then, they are delivered to lysosomes, where they are broken down and disposed. This mechanism aims to get rid of potentially harmful substances or their effects. It is also extensively used during infection [235,236,237,238]. Viruses contain autophagy signalization activators but also can be triggered by coexistent cellular stress or TLRs recognizing pathogen substances [239,240,241]. By the mediation of soluble N-ethylmaleimide-sensitive factor attachment protein receptors (STX17-SNAP29-VAMP8 SNARE), a fusion of autophagosomes and lysosomes occurs. A folding of SNARE complex and later fusion is helped by the multi-subunit homotypic fusion and protein sorting complex (HOPS). It reacts directly with syntaxin-17 (STX17), which is located on an autophagosome [242,243]. Miao et al. have shown that the expression of ORF3a has a negative impact on the creation of the STX17-SNAP29-VAMP8 complex. HOPS subunits, one of which is VPS39, react with ORF3a and are later sequestrated in late endosomes. This reduces the HOPS effect on STX17 and the creation of the SNARE complex, which further reduces the HOPS effect on autophagosomes and lysosomes. Studies show that, through the expression of ORF3a, lysosomes become destabilized [244]. Disrupted lysosome function can result in increased SARS-CoV-2 virus release from the infected cell as viral particles are being released in a lysosomal exocytosis mechanism [245]. This patho-mechanism may be responsible for the infectiousness and pathogenicity of SARS-CoV-2. Overall, the ORF3a effect on VPS39 blocks autophagosome maturation and is responsible for promoting lysosomal exocytosis [246].

Autophagy is also being restricted by ORF7a [247]. Lysosomal enzymes present the highest effectiveness in acidic pH and any changes to the environment where they are active can have detrimental effects on protease activity [248,249]. Koepke et al. showed that ORF7a counteracts the lysosomal environment, becoming acidic. It also reduces the number of acidic lysosomes, which altogether decreases auto-phagosomal degradation. It additionally promotes the exocytosis of viral particles. Researchers have also found out that the closest related bat coronavirus, RaTG13 strain, affects human cells and causes autophagy in the same way and equally well as a human SARS-CoV-2 virus [247]. Similar results, concerning lysosomes, were shown by Ghosh et al. They proved that in cells infected with β-coronavirus, the number of acidic lysosomes decreased, and their pH became higher compared to healthy cells [245]. Increased pH of lysosomes may be caused by high load or defect of proton pump or ion channel [250]. Another mechanism may be the direct act of coronavirus proteins behaving like viroporins, for example, E protein changes the pH of Golgi apparatus from 6.8 to 7.1 [251,252]. Increased pH of lysosomes has a negative impact on the stability and effectiveness of lysosomal enzymes. It defects those organelle functions and the processes they take part in such autophagy, destruction of a pathogen by macrophages, or antigen presentation [253]. Therefore, destroyed lysosomes have a negative impact on antigen presentation and immunologic response, which was shown in studies by Ghosh et al. Their findings point out the fact that a change occurs in antigen presentation from proteins. Macrophages caused higher T lymphocyte activation in peptide presentation compared to cross-presentation. As a result, a change in cross-presentation of antigen from proteins takes place [245].

Blaess et al. showed that, during SARS-CoV-2 infection, substances with lysosome-tropic effect can be used [254]. They group lysosomes independently of the absorption process or their chemical nature. Their concentration inside the organelle can exceed a hundredfold that in the cytoplasm [255,256]. Lysosomo-tropic substances impede infection of cell vesicles and viral reproduction due to cell modifications [254].

Viroporins are selective ion channels necessary for virus replication. They allow ions and some substances to pass through infected cell membranes causing disruption in pH gradients of an organelle. This also affects the Ca^2+^ ions’ flow through the endoplasmic reticulum (ER). Viroporins can be a cause of inflammation reactions by influencing many proteins and intracellular metabolic pathways [257]. They can also take part in the deacidification of the ER-Golgi intermediate compartment (ERGIC) with subsequent growth of cytosol pH [42]. This kind of activity is shown by E protein in SARSCoV-2 [258]. It is found primarily in the Golgi apparatus and ER, where it increases the pH of affected compartments [258]. E protein expression in host cells changes the membrane permeability [259]. Additionally, it creates calcium ion-selective channels and leads to an increased IL-1β production [260,261]. Changes in Ca^2+^ levels affect virus cells as well as host cells, with the latter often undergoing apoptosis after the viroporin expression [262,263].

## 4. Limitations & Future Perspectives

Viral invasion depends on ACE2, TMPRSS2, and CD147 present on the cell membrane. Various small molecules and protein inhibitors have been studied; however, clinical data is still not sufficient to be optimistic about their value. Sample size and insufficient cytokine data have limited numerous clinical trials. Thus, many results should be interpreted cautiously because several studies were discontinued due to the end of COVID-19 outbreaks or were conducted as open-label. Therefore, there is a strong need for more international large-scale, multi-center, randomized trials to fully assess the value and the safety profile of the aforementioned therapeutic agents. Some of these medicines are not affordable worldwide. Agents which are currently in development might be inaccessible to underdeveloped countries. Nevertheless, whereas the pandemic does not respect national boundaries, the distribution of vaccines and novel medicines depends on the wealth of specific societies. Inequality globally poses a risk of other COVID-19 outbreaks.

Nonetheless, impressive research on antiviral therapeutic targets during the COVID-19 pandemic prepared multidisciplinary teams of scientists worldwide for another health crisis requiring instant drug discovery.

## 5. Conclusions

Since the turn of 2019–2020, coronaviral infections have been scrutinized by researchers worldwide. During more than two years of the pandemic, multiple cellular processes disrupted by SARS-CoV-2 have been identified. In this review, we provided an overview of scientific papers on the role of cellular organelles and described promising therapeutic targets. Mechanisms of cell entry were recognized and then targeted by several clinical trials, aiming to milden the course of infection. Moreover, mitochondria might play a significant role in SARS-CoV-2 replication. Mitophagy and autophagy are also disrupted during COVID-19, which leads to the accumulation of malfunctioning organelles. Concluding our survey of cellular dysfunction and host cell organelles during COVID-19, there are still multiple aspects requiring further research in order to understand this complex process better. It is still too soon to predict whether the development of inexpensive agents with a high safety profile is possible during future months.
